# Spontaneous Regression of Optic Disc Pit Maculopathy in a Six-Year-Old Child

**DOI:** 10.4274/tjo.57614

**Published:** 2017-01-17

**Authors:** Sezin Akça Bayar, Almila Sarıgül Sezenöz, Eylem Yaman Pınarcı, Gürsel Yılmaz

**Affiliations:** 1 Başkent University Faculty of Medicine, Department of Ophthalmology, Ankara, Turkey; 2 Başkent University Faculty of Medicine, Department of Ophthalmology, İstanbul, Turkey

**Keywords:** Optic pit maculopathy, optic pit, spontaneous regression

## Abstract

A 6-year-old boy with a complaint of blurred vision for two months was referred to our clinic. His visual acuity was 20/32 in the right eye and 20/20 in the left eye. Optical coherence tomography (OCT) revealed optic disc pit maculopathy in the right eye. The patient was followed for 6 months without any treatment. At the end of the 6-month period, the patient’s visual acuity was 20/20 in both eyes. The OCT imaging showed spontaneous regression of the optic disc pit maculopathy. In this case report, it is concluded that in children, spontaneous regression of the optic pit maculopathy with full recovery of visual acuity is possible. The development of optic pit maculopathy in childhood is rare and there are not enough studies on the treatment methods. Therefore, our case report may be helpful in the management of similar cases of pediatric optic disc maculopathy.

## INTRODUCTION

Optic disc pit (ODP) is a rare congenital optic disk abnormality with an incidence of 1/11,000. ODPs are hypopigmented, yellowish, gray-white, oval or round depressions that are usually located unilaterally at the temporal part of the optic disc.^1,2,3^ ODPs are usually asymptomatic and noticed during routine eye examinations. However, some patients with ODP demonstrate significant macular changes, resulting in irreversible central visual field defects and reduced central visual acuity. These macular changes, including serous macular detachment, cystic degeneration, and degenerative pigment changes, are defined as ODP-induced maculopathy (ODP-M).^1^ The great majority of ODP-Ms become symptomatic in the third or fourth decade of life.^1^ Twenty-five percent of ODP-Ms resolve spontaneously, but the final outcomes of these cases are shown to be poor.^1^ Therefore, different treatment modalities such as vitreoretinal surgery or laser photocoagulation may be preferable over conservative management. In the literature, there are only a few cases of childhood-onset ODP-M that showed spontaneous resolution.^2,4,5^ In this report we present a case of a 6-year-old-boy with ODP-M. To our knowledge, this is the first case of ODP-M in children which spontaneously regressed with full recovery of visual acuity.

## CASE REPORT

A previously healthy 6-year-old boy who had a complaint of blurry vision in his right eye for two months was referred to the retina department. His best-corrected visual acuity (BCVA) measured with Snellen chart was 20/32 in the right eye and 20/20 in the left eye. Anterior segment findings and intraocular pressures of both eyes were normal. Refraction results were +0.75 D in the right eye, and +0.50 D in the left eye. On fundoscopic examination a temporally located ODP associated with cystic changes in the macular area was detected in the right eye, while no pathological changes were seen in the left eye (Figure 1A). High-resolution (HR) optical coherence tomography (OCT) (Cirrus, Carl Zeiss Meditec. Inc.) imaging revealed a schisis cavity due to the optic pit and cystoid changes due to fluid collection under the internal limiting membrane in the right eye (Figure 1B). The patient had no family history or known macular disorder. As the patient’s visual acuity was 20/32, we opted for a conservative approach and the patient was followed for 6 months without any treatment. In the ophthalmologic examination at the end of the 6-month period, the patient’s BCVA was 20/20 in both eyes. The maculopathy had resolved, leaving some residual pigment epithelial changes (Figure 2A). OCT images showed regression of the previous findings of ODP-M in the right eye (Figure 2B).

## DISCUSSION

Although spontaneous resolution of ODP-M with improvement of visual acuity has been reported, the prognosis remains poor in approximately 25% to 75% of the patients.^2,4^ A few cases of spontaneous regression of ODP-M in children have been previously reported in the literature.^2,4,5^ Yuen and Kaye^2^ noted a case with spontaneous resolution in which the visual acuity of an 8-year-old patient improved from light perception to 2/30. Sugar^4^ reported a 4-year-old child whose vision recovered after spontaneous regression of the subretinal fluid within 18 months. Schatz and McDonald^5^ reported a 6-year-old child with spontaneous near-complete reattachment of the macula within 5 months. In these studies, conservative management was preferred as 25% of ODP-Ms resolve spontaneously.^2,4^

In our case, visual improvement started within 3 months. At the end of the sixth month, unlike other cases in the literature, full anatomical and functional recovery was obtained without any need for additional interventions.

Though not seen in our case, fluid accumulation between retinal layers is a common finding in ODP-M. In a recent study of 16 patients with ODP, it was shown by HR-OCT that fluid can move directly from the optic pit to the subinternal limiting membrane space, ganglion cell layer, inner and outer nuclear layers, or subretinal space.^6^ In our case, HR-OCT revealed a schisis cavity due to the optic pit and cystoid changes due to fluid accumulation under the internal limiting membrane. Despite numerous case reports offering different treatment modalities, the best method has not yet been determined for pediatric cases. In previous years, laser photocoagulation was used in cases that did not show spontaneous improvement within three months. Now, however, the presence of vitreous traction is the most important determining factor in treatment decisions. Most retina specialists recommend the combined use of laser photocoagulation of the peripapillary region and vitrectomy with or without ILM-peeling.^1,7^ However, particularly in pediatric cases, 3-6 months of follow-up before any surgical and invasive procedures is appropriate.

## CONCLUSION

In the management of ODP-M cases, it must be kept in mind that spontaneous regression is possible, especially in pediatric cases such as ours. Therefore, a more conservative approach may be beneficial in the treatment of young patients.

### Ethics

Peer-review: Externally peer-reviewed.

## Figures and Tables

**Figure 1A:**
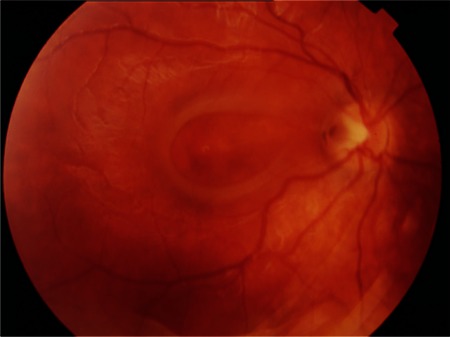
Fundus photograph of the right eye showing the optic disc pit and maculopathy

**Figure 1B:**
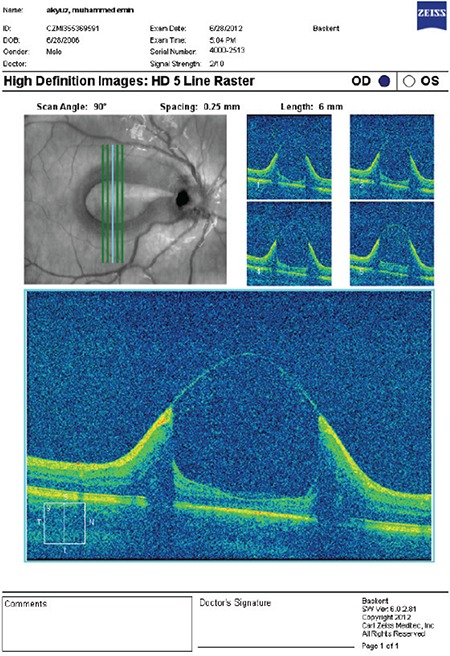
Optical coherence tomography image of the macula of the right eye taken at presentation

**Figure 2A:**
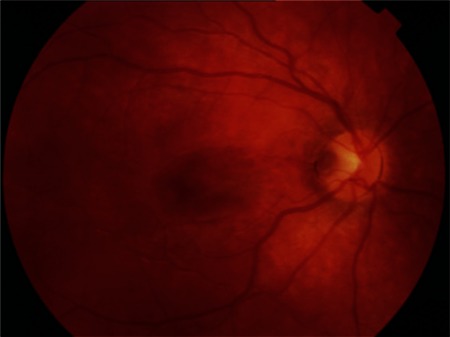
Fundus photoghraph of the right eye showing resolution of the optic disc pit maculopathy

**Figure 2B:**
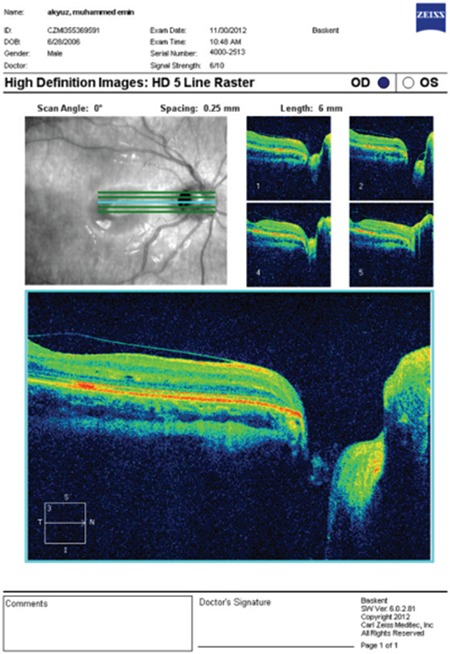
Optical coherence tomography image of the right eye taken at final examination
